# Epoxyeicosatrienoic acid analog EET-B attenuates post-myocardial infarction remodeling in spontaneously hypertensive rats

**DOI:** 10.1042/CS20180728

**Published:** 2019-04-29

**Authors:** Jan Neckář, Md. Abdul Hye Khan, Garrett J. Gross, Michaela Cyprová, Jaroslav Hrdlička, Alena Kvasilová, John R. Falck, William B. Campbell, Lenka Sedláková, Šárka Škutová, Veronika Olejničková, Martina Gregorovičová, David Sedmera, František Kolář, John D. Imig

**Affiliations:** 1 Department of Pharmacology & Toxicology, Medical College of Wisconsin, Milwaukee, WI, USA; 2 Department of Developmental Cardiology, Institute of Physiology of the Czech Academy of Sciences, Prague, Czech Republic; 3 Center for Experimental Medicine, Institute for Clinical and Experimental Medicine Physiology, Prague, Czech Republic; 4 Institute of Anatomy, First Faculty of Medicine, Charles University, Prague, Czech Republic; 5 Department of Biochemistry, University of Texas Southwestern Medical Center, Dallas, TX, USA

**Keywords:** spontaneously hypertensive rat, myocardial infarction, blood pressure, congestive heart failure, epoxyeicosatrienoic acid

## Abstract

Epoxyeicosatrienoic acids (EETs) and their synthetic analogs have cardiovascular protective effects. Here, we investigated the action of a novel EET analog EET-B on the progression of post-myocardial infarction (MI) heart failure in spontaneously hypertensive rats (SHR). Adult male SHR were divided into vehicle- and EET-B (10 mg/kg/day; p.o., 9 weeks)-treated groups. After two weeks of treatment, rats were subjected to 30-min left coronary artery occlusion or sham operation. Systolic blood pressure and echocardiography measurements were performed at the beginning of study, 4 days before, and 7 weeks after MI. At the end of the study, tissue samples were collected for histological and biochemical analyses. We demonstrated that EET-B treatment did not affect blood pressure and cardiac parameters in SHR prior to MI. MI was induced and cardiac parameters were measured 7 weeks later. Fractional shortening (FS) was decreased to 18.4±1.0% in vehicle-treated MI rats compared with corresponding sham (30.6±1.0%) 7 weeks following MI induction. In infarcted SHR hearts, EET-B treatment improved FS (23.7±0.7%), markedly increased heme oxygenase-1 immunopositivity in cardiomyocytes and reduced cardiac inflammation and fibrosis (by 13% and 19%, respectively). In conclusion, these findings suggest that EET analog EET-B has beneficial therapeutic actions to reduce cardiac remodeling in SHR subjected to MI.

## INTRODUCTION

Congestive heart failure (CHF) is a leading cause of morbidity and mortality that created major healthcare and economic burden in the Western world. In most instances, CHF is the irreversible and final consequence of a variety of etiologies; however, a predominant cause is coronary heart disease [1]. Several factors such as hypertension, diabetes mellitus or obesity contribute to CHF development and progression as well as organ damage to lung and kidneys. Although considerable progress has been made in CHF management, novel therapeutic strategies are required.

Epoxyeicosatrienoic acids (EETs), cytochrome P450 epoxygenase metabolites of arachidonic acid, represent a promising pharmacological approach in cardiovascular disease prevention. A large body of evidence indicates that EETs are important paracrine agents with robust organ protective actions. EETs prevent various cardiovascular diseases, due to their vasodilator, anti-hypertensive, anti-inflammatory and other beneficial biological actions [2,3]. The main limitation for EETs as a therapeutic application is their short half-life due to EETs being quickly converted to biologically inactive dihydroxyeicosatrienoic acids (DHETs) by the enzyme soluble epoxide hydrolase (sEH). Considering these facts, it is conceivable that any effective EETs therapeutic utilization for cardiovascular diseases can only be achieved by two major approaches.

The first approach is based on pharmacological blockade of sEH to reduce the degradation of EETs to DHETs. Previously, we and others established that the acute administration of sEH inhibitors improved cardiac ischemic tolerance in normotensive and hypertensive animals [4–7]. Moreover, it was determined that chronic pharmacological intervention with sEH inhibitors reduced myocardial hypertrophy and fibrosis development and progression, decreased inflammation and improved heart function post-myocardial infarction (MI) [8–11].

A second approach involves the use of agonistic EET analogs with markedly longer (hours) half-life. The primary advantage of this strategy is that EET analogs are biologically more stable than endogenously produced EETs. Hence, several EET analogs have been developed and demonstrated powerful protective actions against several cardiovascular diseases [12–20]. Here, cardioprotective effects of a novel, stable and orally active agonistic 14,15-EET analog EET-B [15] was examined in spontaneously hypertensive rats (SHR), a pre-clinical rodent model of human essential hypertension [21]. SHR were subjected to MI and the effects of continuous EET-B treatment before and after MI on post-ischemic left ventricular (LV) function, myocardial fibrosis and inflammation were analyzed. As EET-based therapies can attenuate the progression of CHF by mechanisms involving activation of heme oxygenase-1 (HO-1) [22]. HO-1 immunopositivity in viable myocytes of the ischemic myocardium was also determined. Our findings demonstrate that EET-B treatment has beneficial actions on the SHR heart following MI.

## METHODS

### Animal groups and experimental protocol

**Figure 1:**
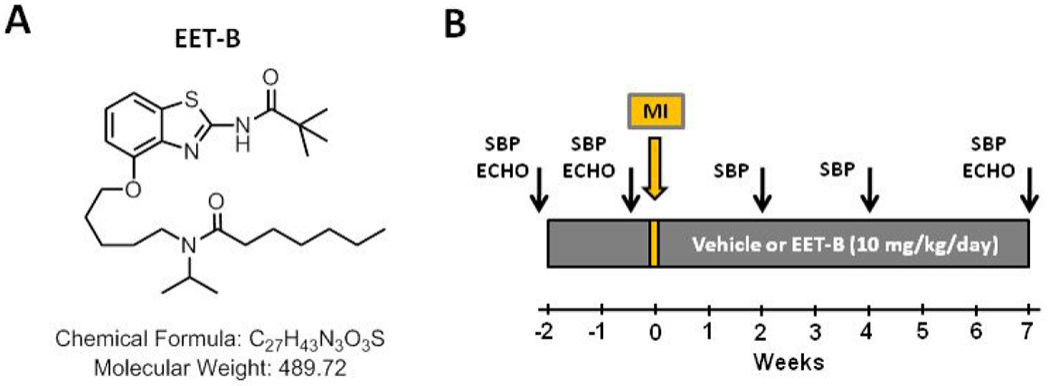
Structure of EET-B [*N*-isopropyl-*N*-(5-((2-pivalamidobenzo[*d*]thiazol-4-yl)oxy)pentyl) heptanamide] **(A)**. The design of the experiments analyzing the cardio-protective action of EET-B in rat hearts **(B)**. Systolic blood pressure (SBP) and echocardiography (ECHO) assessment of left ventricular function and geometry were performed at the start of the study, before MI and during 7 weeks of reperfusion. See Materials and Methods for the detailed description.

The EET analog EET-B |*N*-isopropyl-*N*-(5-((2-pivalamidobenzo[*d*]thiazol-4-yl)oxy)pentyl) heptanamide] (Figure 1A) was designed and synthesized in the laboratory of John R. Falck. Pharmacokinetic studies analyzing oral administration of EET-B predicted its half-life at 10-15 hrs [23,24]. The EET-B dose was based on our previously published studies that determined the dose-response for vascular reactivity and showed antihypertensive and renal protective effects in salt-sensitive and angiotensin II-dependent hypertension [15,16,25].

The Medical College of Wisconsin Institutional Animal Care and Use Committee according to the National Institutes of Health Guidelines for Care and Use of Laboratory Animals approved all animal studies. Ten-week old male spontaneously hypertensive rats (SHR) were purchased from Charles River Laboratories (Wilmington, MA, USA). Animals were housed in the Biomedical Resource Center at Medical College of Wisconsin with a 12/12h light–dark cycle and free access to tap water and rodent chow. SHR were divided into two groups. The first group received vehicle (0.05% ethanol and 0.1% PEG-400), while the second group received EET-B (10 mg/kg/day; p.o.) from 2 weeks before MI till 7 weeks thereafter.

SHR were weighed and systolic blood pressure (SBP) was measured by tail-cuff plethysmography (IITC Life Science Inc., Woodland Hills, CA) at the beginning of study, 4 days before and 2, 4 and 7 weeks after MI (Figure 1B). Rats were trained for blood pressure measurement at the same time of the day (9 – 12 am) for 3 days before every intervention.

### Echocardiographic assessment of cardiac function

Echocardiography was carried out to determine function of the left ventricle (LV) by an ultrasound-based noninvasive technique (Transthoracic Echocardiography, Visual Sonics Vevo 770 with RMV 710B probe, Toronto, Canada). Animals were anesthetized with 2% isoflurane (Forane; Abbott Laboratories, Queenborough, UK) mixed with room air. Prior to starting the treatment protocol, echocardiography measurements were performed at baseline and then 4 days before and 7 wks after myocardial infarction (MI) or sham operation in anesthetized rats (Figure 1B). Diastolic and systolic dimensions of left ventricle (LV) were measured during echocardiography evaluation including anterior and posterior wall thickness (AWTd, PWTd, AWTs, PWTs) and LV cavity diameter (LVDd, LVDs). From these dimensions, the following functional echocardiography parameters were derived: fractional shortening (FS) using the formula (LVDd-LVDs)/LVDd*100, systolic thickening of anterior wall (ΔAWT) from the equation (AWTs-AWTd)/AWTd* 100, and relative wall thickness from the equation (AWTd-PWTd)/LVDd*100.

### Myocardial ischemia

After the second echocardiography evaluation (before MI), Vehicle- or EET-B-treated SHR were divided into four groups: i) Sham-Vehicle, ii) MI-Vehicle, iii) Sham-EET-B, iv) MI-EET-B. Anesthetized (sodium pentobarbital, 60 mg/kg i.p.) open-chest rats ventilated with room air (1.2 ml/kg) were subjected to 30 min of left anterior descending (LAD) coronary artery occlusion or sham surgery as described previously [26]. At the start of reperfusion, the chest was closed, air was removed from the thorax and spontaneously breathing animals recovering from anesthesia were housed in separate cages and received analgesia (Buprenorphine, 0.5 – 1 mg/kg s.c.) followed for another two days. The incidence and severity of ventricular arrhythmias during the 30-min ischemic insult were assessed according to the Lambeth Convention as previously described [27]. Premature ventricular complexes (PVCs) occurring as singles, salvos or tachycardia (VT, a run of 4 or more consecutive PVCs) were counted separately. The incidence and duration of life-threatening ventricular tachyarrhythmias, i.e. VT and fibrillation (VF), were also evaluated. VF lasting more than 2 min was considered as sustained (VFs). Rats exhibiting VFs were excluded from further evaluation.

### Histopathological studies

The day after the final echocardiography measurements, rats were euthanized by cervical dislocation and hearts were collected for histological analysis. Hearts were excised, washed with Tyrode’s solution, perfusion-fixed and stored in 4 % paraformaldehyde for 2 days at 4° C. Hearts were cut perpendicularly to the long axis at the largest circumference and embedded in paraffin. Tissue sections were cut into 4 *μ*m slices for use in histology protocols. Formalin-fixed and paraffin-embedded tissue slices were de-paraffinized, rehydrated and stained with Hematoxylin-Eosin or Picro-Sirus Red staining for gross histopathological examination and determination of collagen positive fibrotic areas in the heart. Tissue sections were also immunostained with anti-HO-1 (1:1000, Abcam, Cambridge, UK). Horseradish peroxidase-conjugated goat anti-rabbit secondary antibody (1:200; Jackson ImmunoResearch Laboratories, west Grove, PA, USA) was used for development with avidin-biotinylated HRP complex (Vectastain ABC Elite kit, Vector Laboratories, Burlingame, CA, USA) followed by counterstaining with hematoxylin. Stained sections were visualized by light microscopy at 400× magnification and digital images of the stained sections were taken for analysis using Nikon NIS Elements Software (Nikon Instruments Inc., Melville, NY, USA). Collagen content and HO-1 expression were determined by quantification of Picro-Sirus Red stained and HO-1-positive areas in viable cardiomyocytes, respectively (10-15 images for each heart sections) by two experienced observers in a blinded fashion.

### Statistical analysis

The results are expressed as means ± SEM. Unpaired Student’s t-test or one-way ANOVA and subsequent Student-Newman-Keuls test were used for comparison of differences in normally distributed variables between groups. Not normally distributed data (arrhythmias) were expressed as median (range). Differences in the number of premature ventricular complexes between the groups were compared by Mann-Whitney test. The incidence of tachycardia and fibrillation and mortality were examined by Fischer’s exact test. Differences were considered statistically significant when *P*<0.05.

## RESULTS

### EET-B treatment did not affect blood pressure and body weight

**Figure 2:**
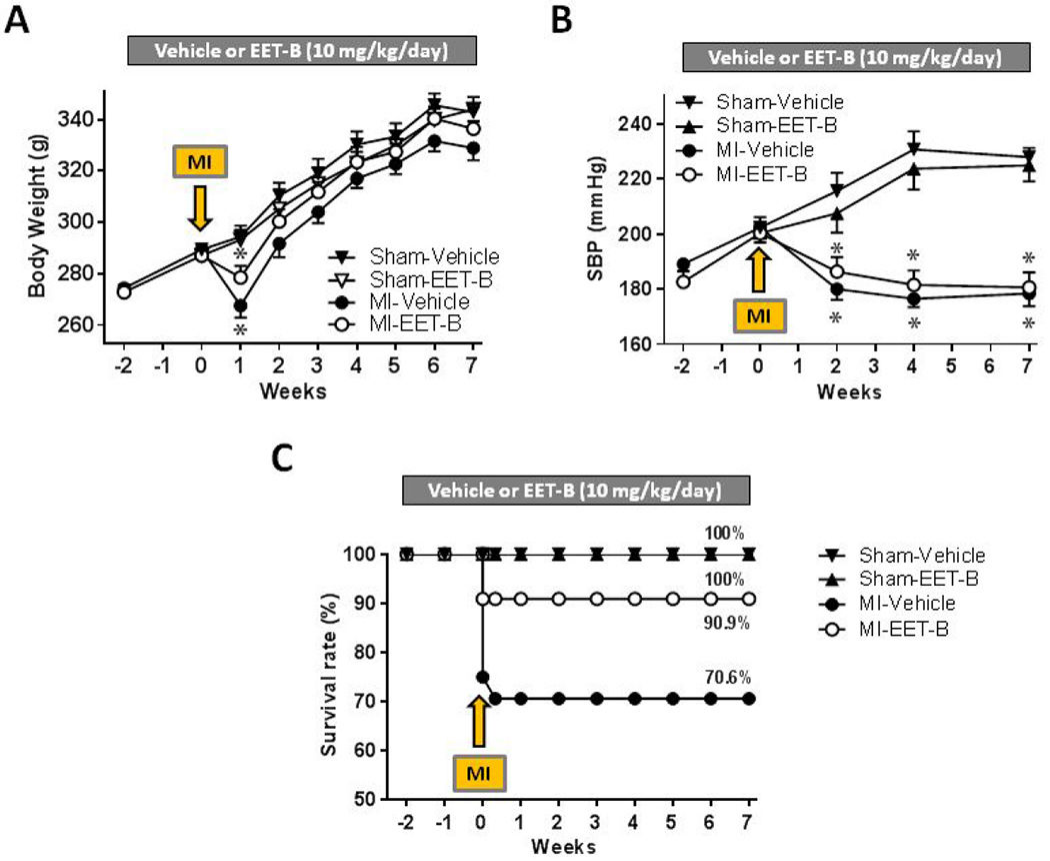
Body weight **(A)**, systolic blood pressure (SBP) **(B)**, and survival rate **(C)** in vehicle- and EET-B-treated SHR subjected to myocardial infarction (MI) and Sham-operated groups. Rats were treated by vehicle or EET-B (10 mg/kg/day) from two weeks before till seven weeks after MI. Values are means ± SEM from 6-12 rats in each group. *P<0.05 vs. corresponding Sham-operated group.

At the beginning of the study, the experimental groups did not differ in SBP and body weight (BW; Figure 2A,B). In both MI groups, a significant reduction in BW was evident one week after MI. However, from the second week post-MI, weight gain occurred in the groups subjected to ischemia, and by the end of the study protocol BW was similar among all groups (Figure 2A). Surprisingly, EET-B pretreatment to SHR prior to MI had no effect on SBP (Figure 2B). MI significantly reduced SBP by 35 – 45 mmHg seven weeks following MI due to heart failure in both vehicle- and EET-B-treated groups compared to corresponding sham-operated rats.

### EET-B treatment reduced ischemic arrhythmias and early post-MI mortality

Sustained VF occurred in one vehicle-treated MI SHR. Four vehicle-treated and one EET-B-treated SHR died in the early post-ischemic period (within 48 hrs. after MI). Therefore, ischemic and early reperfusion mortality reached 29.4% and 9.1%, respectively, in vehicle- and EET-B-treated SHR and did not increase during 7 week follow up post-MI period (Figure 2C). Interestingly, EET-B-treated SHR exhibited lower incidence and severity of ischemic ventricular arrhythmias compared to vehicle-treated SHR but the differences did not reach statistical significance (Table 1).

### EET-B treatment attenuated the progression of post-MI heart failure

**Figure 3:**
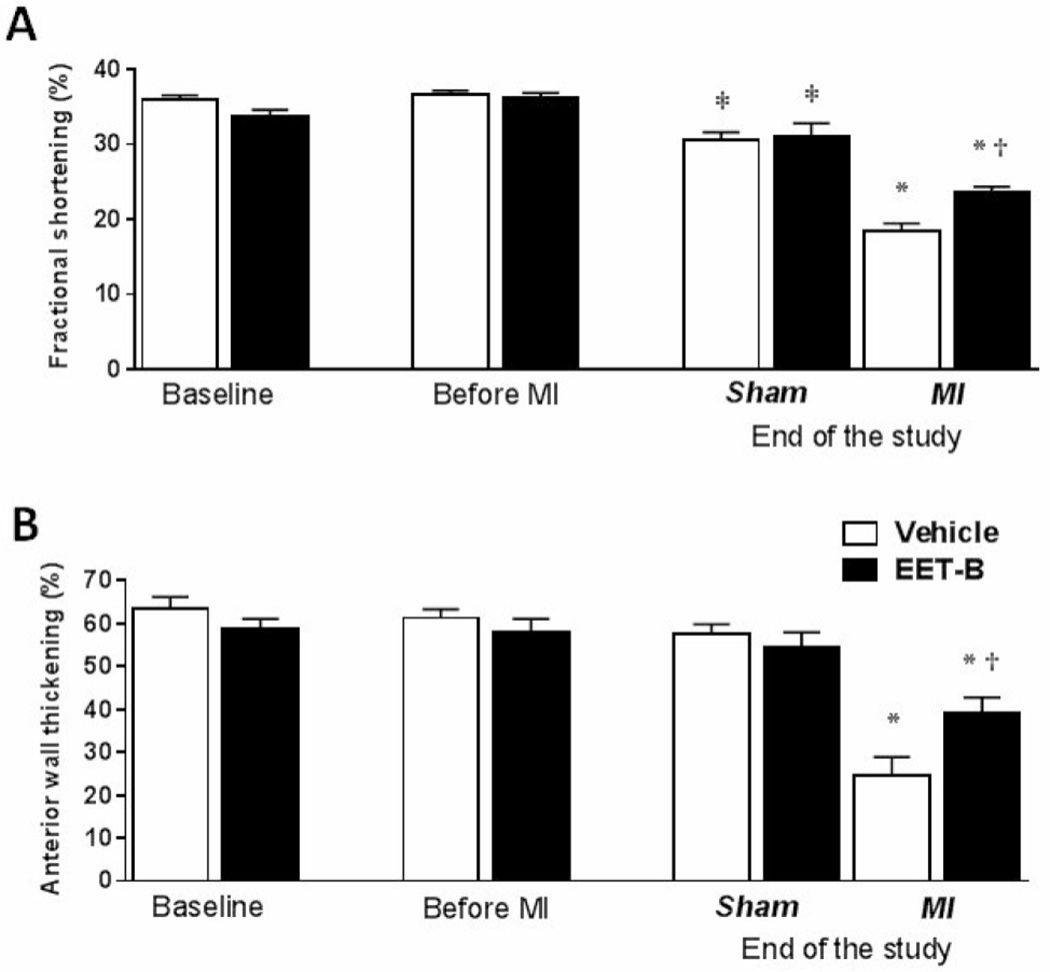
Echocardiography assessment of left ventricular fractional shortening **(A)** and the anterior wall systolic thickening **(B)** in vehicle- and EET-B-treated SHR subjected to myocardial infarction (MI) and Sham-operated groups at the start of study (Baseline), after 10 days of treatment (Before MI) and 7 wks after MI (End of the study). Rats were treated by vehicle or EET-B from two weeks before till seven weeks after MI. Values are means ± SEM from 6-12 rats in each group. *P<0.05 vs. corresponding Sham-operated group; ^†^*P*<0.05 vs. MI-Vehicle group; ^‡^*P*<0.05 vs. corresponding Before MI group.

As summarized in Figure 3 and Table 2, LV systolic function and geometry assessed by echocardiography were not altered by vehicle or EET-B treatment prior to MI. A significant increase in systolic and diastolic LV diameters were found in sham vehicle- and EET-B-treated controls at the end of study. The observed dilation of the LV chamber in both sham-operated groups reflected a significantly lower FS (by 16.3% and 14.2%, respectively) compared to values prior to surgery (Figure 3A). In the SHR group subjected to MI and vehicle-treated, both FS and ΔAWT were markedly decreased to 18.4±1.0% and 24.5±4.4%, respectively, compared to age-matched sham-operated controls that averaged 30.6±1.0% and 57.5±2.6%, respectively. EET-B treatment significantly attenuated MI-induced reductions in FS and ΔAWT to 23.7±0.7% and 39.1±3.6%, respectively (Figure 3).

The relative lungs weight significantly increased by 75.4% in the Mi-vehicle group compared with corresponding sham-operated controls. In MI-EET-B group, the lungs weight increase was significantly less pronounced (31.1%) suggesting that EET-B treatment attenuated the progression of CHF-induced lung edema (Table 3). With respect to heart weight (HW), MI increased HW/BW ratio by 10% and 13%, respectively, compared to corresponding sham-operated groups (Table 3).

### EET-B treatment reduced post-MI fibrosis and inflammation

**Figure 4:**
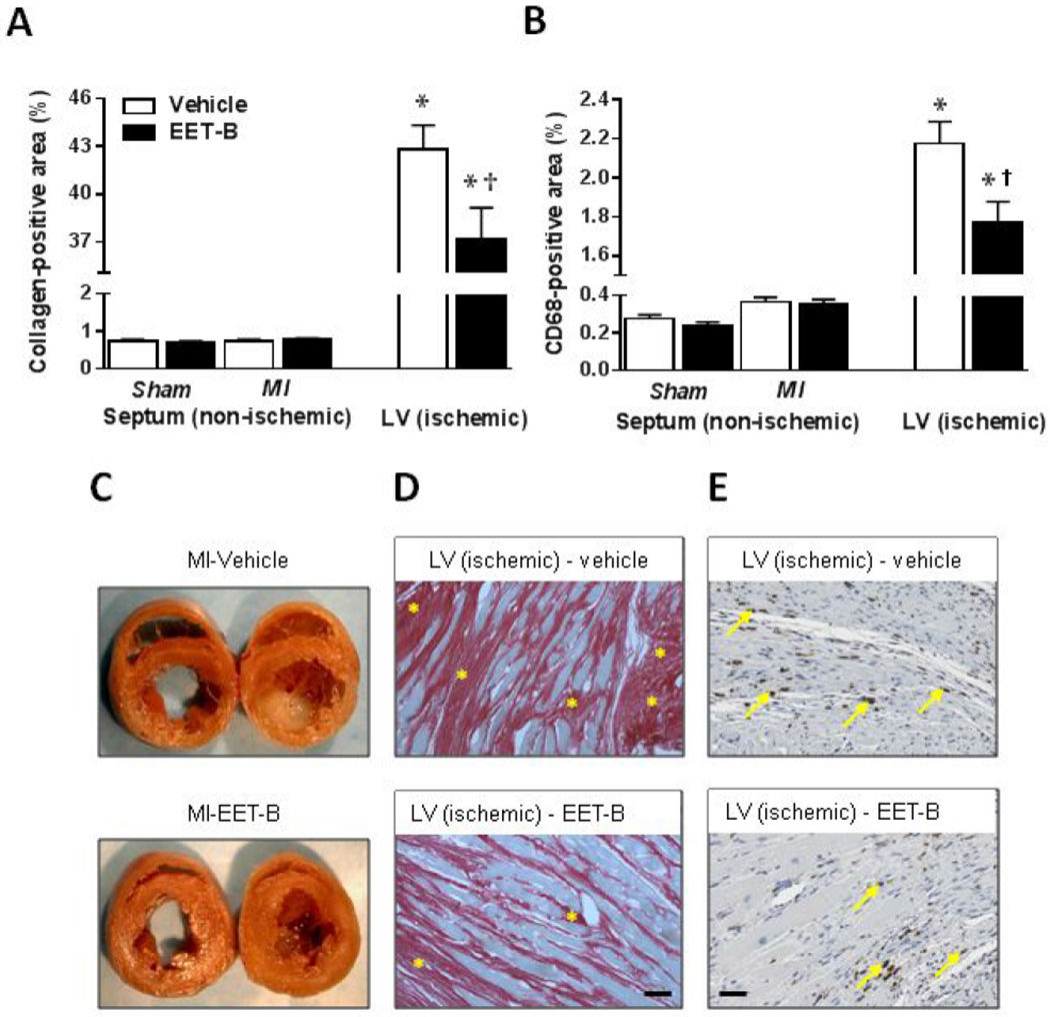
Quantification of myocardial collagen-positive area **(A)** and CD68-positive area **(B)** assessed in non-ischemic septum, and ischemic area of left ventricle (LV) of vehicle- and EET-B-treated SHR subjected to myocardial infarction (MI) and Sham-operated groups. Macroscopic images of heart cross-sections **(C)**, photomicrographs of Picro-Sirius Red staining of collagen positive areas (carmine red marked by stars) **(D),** and immunohistochemical staining depicting macrophage/monocyte (CD68-positive; dark brown dots marked by arrows) **(E)**. Rats were treated by vehicle or EET-B from two weeks before till seven weeks after MI. Scale bars represent 50 μm. Values are means ± SEM from 6-10 rats in each group. *P<0.05 vs. Sham-Vehicle group. ^†^*P*<0.05 vs. MI-Vehicle group.

In the vehicle-treated SHR ischemic area, collagen fraction as determined by Picro-Sirius Red staining and monocytes/macrophages infiltration as determined from the presence of CD68 positive cells reached 42.8±1.5% and 2.18±0.11%, respectively (Figure 4). EET-B treatment significantly reduced myocardial fibrosis and CD68 positive area to 37.2±1.9% and 1.77±0.11%, respectively. In non-ischemic septum of vehicle- or EET-B-treated SHR subjected to MI or Sham operation, collagen content as well as monocytes/macrophages infiltration did not differ among the groups (Figure 4).

### EET-B treatment increased HO-1 immunopositivity in viable LV cardiomyocytes post-MI

**Figure 5:**
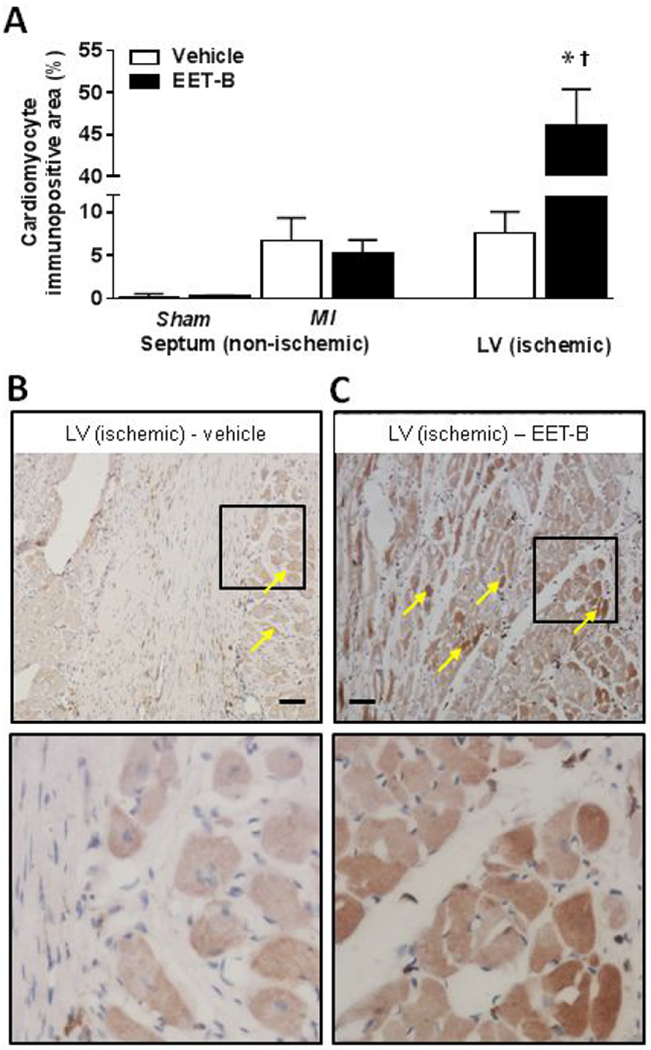
Quantification of heme oxygenase-1 (HO-1) immunopositivity in viable cardiomyocytes assessed in non-ischemic septum, and ischemic area of left ventricle (LV) of vehicle- and EET-B-treated SHR subjected to myocardial infarction (MI) and sham-operated groups **(A)**, photomicrographs of immunohistochemical staining depicting HO-1 in ischemic area of LV (marked by arrows) in vehicle- **(B)** and EET-B-treated (**C**) rats. The lower pictures show the square areas in higher magnification. Rats were treated by vehicle or EET-B from two weeks before till seven weeks after MI. Scale bars represent 75 μm. Values are means ± SEM from 6-10 rats in each group. *P<0.05 vs. Sham-Vehicle group. ^†^*P*<0.05 vs. MI-Vehicle group.

In both sham-operated SHR groups (vehicle- and EET-B-treated), HO-1 immunopositivity in cardiomyocytes was low and averaged 0.20±0.08% and 0.25±0.11%, respectively (Figure 5A). MI increased HO-1 cell immunopositivity in non-ischemic septa in both vehicle- and EET-B-treated SHR (Figure 5). This finding suggests that post-MI cardiac remodeling could activate HO-1-mediated cell signaling. Moreover, EET-B treatment markedly increased HO-1 immunopositivity in viable cardiomyocytes in the infarcted area to 46.0±4.4% (Figure 5).

## DISCUSSION

The main finding of the present study is that the orally active EET analog, EET-B reduced post-MI mortality and systolic dysfunction progression in SHR. Cardio-protective actions for EET-B were associated with diminished CHF-induced lung edema, reduced myocardial fibrosis, decreased monocytes/macrophages infiltration in the ischemic area, and increased HO-1 immunopositivity in viable cardiomyocytes after MI. Taken together, we demonstrate that EET-B attenuates CHF progression without altering blood pressure in SHR with established hypertension.

It is well known that EETs possess multiple biological functions including vasodilation, anti-inflammatory, anti-fibrotic, and also affects lipid metabolism and insulin sensitivity [2,9,28–30]. EETs have been demonstrated to contribute to neovascularization by promoting angiogenesis [31,32]. In the heart, the acute administration of EETs or sEH inhibitors prior to ischemia and at the start of reperfusion increased cardiac ischemic tolerance in several animal species and experimental settings [4,6,7,33,34]. Recently, cardio-protective actions for acute administration of EET analogs against ischemia/reperfusion injury have been determined. Indeed, UA-8, a dual acting compound possessing EET mimetic and sEH inhibitory properties, improved cardiac ischemic tolerance in isolated mouse hearts [35]. Likewise, EET-A, another orally active EET analog with similar protective potential to EET-B [36], reduced myocardial infarction in normotensive Sprague-Dawley rats [12]. Finally, in a recent study we revealed a potent infarct size-limiting effect of acute EET-B administration given at the start of reperfusion in normotensive rats [14]. We further demonstrated that EET-B provided cardio-protection comparable to native 14,15-EET, and its protective action was completely abolished by co-administration with 14,15-EEZE, the selective antagonist of 14,15-EET [14]. These findings clearly demonstrated that EET-B is an effective agonistic 14,15-EET analog.

Previous experimental studies demonstrated that chronic treatment with sEH inhibitors results in increased EET levels and diminishes the development of LV hypertrophy due to pressure or volume overload [19,37–42]. We reported that two-week treatment with orally active EET-analogs attenuate hypertension-induced organ injury by reducing inflammation, oxidative stress, and endoplasmic reticulum stress [15,16]. One orally active EET analog, EET-A reduced the incidence of life-threating ischemic arrhythmias in transgenic *Ren-2* rats with angiotensin II-dependent hypertension [19] which is consistent with the anti-arrhythmic action of EET-B observed in the present study.

Interestingly, the cardio-protective effects for EET-B in the present study were independent of blood pressure independent. The hypertension severity before MI and the reduction of SBP due to heart failure were similar in vehicle- and EET-B-treated SHR. Blood pressure-independent kidney and myocardial protective actions for EET-B have been reported also in Dahl salt-sensitive (SS) hypertensive rats [15]. The inability for EET-B to exert a blood pressure independent action in Dahl SS rats and in SHR post-MI could be related to the fact that, unlike native EETs, EET-B lacks a natriuretic effect [15]. However, based on previous evidence for blood pressure independent organ protective actions with sEH inhibitor administration in SHR [43], we cannot exclude that SHR, a model of essential hypertension, or other hypertensive rat models with a different genetic background could be less sensitive to anti-hypertensive EET-based therapy than that consistently observed in angiotensin II-dependent hypertension [16,19,33,44]. In any case, our findings of the present study clearly indicate that EET-B had blood pressure independent actions to improve heart function in SHR with CHF.

Coronary artery disease is the main cause of CHF development [1] and has a very poor prognosis [45]. Therefore, determining pathophysiological mechanisms underlying post-MI cardiac remodeling and development of new therapeutic approaches are needed. In the present study, we investigated cardioprotective ability of EET-B. However, it should be noted that the published findings are not completely clear with respect to EET cardio-protective actions on the progression of heart dysfunction in humans after MI. Monti et al. [40] reported lower plasma 14,15-DHET levels and decreased *EPHX2*, the gene coding sEH, in myocardial biopsies harvested at the time of cardiac surgery in patients with severe CHF. This finding was interpreted as an adaptive transcriptional process aimed at maintaining high EET levels. Zhang et al. [46] observed attenuation of 14,15-DHET plasma levels in cardio-renal disease patients with decreased cardiac function and increased risk of adverse myocardial events. Schuck et al. [47] demonstrated an inverse association between coronary artery disease severity and the plasma 14,15-EET/DHET ratio, a biomarker for sEH activity. Finally, it has been reported that an sEH inhibitor increased EET levels in endothelial progenitor cells (important for vasculogenesis and re-endothelization), that are collected from patients following acute MI [29].

Unlike human, animal studies on the cardio-protective potential for EET-based therapy in failing hearts after MI are more consistent. It has been shown that NUDSA, an earlier generation EET analog, improved cardiac function in mice after MI [18]. Moreover, an attenuation of post-MI LV dysfunction progression has been reported in rats and mice treated with various sEH inhibitors [8,10,11,37,48]. In line with these earlier findings, we demonstrated that EET-B treatment (started two weeks before MI and continued till seven weeks after MI) attenuated post-MI LV systolic dysfunction and adverse changes of ventricular geometry in the present study. This EET-B treatment prior to ischemia in SHR could have additional effects on acute cardiac ischemic tolerance as suggested by the reduction of life-threating arrhythmias [19]. In addition, the improved post-MI LV function is more likely attributable to EET-B beneficial actions during CHF progression as it was previously demonstrated for other EET-based pharmacological interventions [8,10,11,18,37,48]. Finally, in a recent work we demonstrated that sEH inhibitor c-AUCB and EET analog EET-A, given alone or in combination at reperfusion attenuated the progression of post-MI LV systolic dysfunction only in Sprague-Dawley but not in hypertensive *Ren-2* transgenic rats in spite of blood pressure reduction [19,49]. Moreover, these findings support previous findings indicating that the protective action of EET analogs against various cardiovascular diseases is independent of their antihypertensive actions.

The effect of EET-based therapy on the progression of CHF-associated etiologies other than ischemic heart disease is more inconsistent. Indeed, it has been reported that sEH inhibitors can reduce [11,42] or unchanged [50] the development of cardiac hypertrophy and diminish adverse cardiac remodeling in normotensive mice and rats subjected to pressure overload. Similarly, sEH inhibitors did not alter LV contractility in normotensive and hypertensive rats subjected to CHF induced by volume overload [51–53].

In the current study, EET-B treatment in SHR subjected to MI decreased cardiac fibrosis that resulted in improved FS and ΔAWT compared to vehicle-treated SHR seven weeks following MI. Pathological remodeling and cardiac fibrosis are strongly associated with inflammation that contributes to CHF progression. Earlier studies in rodents clearly demonstrated protective effects for EET-based therapy in the cardiac fibrosis prevention after MI [8,10,11,18,48] and in hearts subjected to pressure overload [9,11,54,55]. All these findings support the idea that increased endogenous EET levels, as well as, EET analogs provide beneficial anti-fibrotic and anti-remodeling actions in the injured myocardium. It should be noted that among the approaches to increase endogenous EET levels and/or increase EET bioavailability by sEH inhibition, only pharmacological sEH inhibition seems to be effective. Indeed, sEH gene deletion (*EPHX2*^−/−^) did not show any positive effect in an experimental setting of pathological remodeling and cardiac fibrosis after angiotensin II treatment [9], and, unexpectedly, increased mortality in mice due to cardiac arrest [56].

Another potential mechanism for EET-B treatment to attenuate CHF progression in SHR could be through anti-inflammatory actions. Previous studies revealed that sEH inhibitors reduce myocardial inflammation after MI [8,9]. Most importantly, we showed marked anti-inflammatory actions of EET-B associated with its robust kidney protective ability in rat models of salt-sensitive [15] and angiotensin-II dependent hypertension [44]. In this study, we demonstrated monocytes/macrophage infiltration into the ischemic area in vehicle-treated SHR with CHF, and it was reduced by EET-B treatment. Taken together, in post-MI SHR, EET-B mediated improvement of LV systolic function accompanied by reduction in cardiac fibrosis and inflammation.

EETs ability to interact with HO-1 is another apparent mechanism that has been attributed to organ protective action [57,58]. We examined role of HO-1 in EET-B cardio-protective actions in Mi-induced CHF in SHR. Our findings revealed that EET-B increased HO-1 level in viable LV myocytes of SHR subjected to MI. This finding supports previous experimental findings that EET induction of HO-1 is an essential event in protecting myocardium against acute ischemia/reperfusion injury and the progression to post-ischemic CHF [57,59]. Cao et al. [18] showed that EET analog NUDSA increased HO-1 protein level which was associated with improved LV systolic function and reduced collagen fraction in the scar of post-MI mouse hearts, and co-treatment with an HO activity inhibitor reversed this effect. In SHR, pharmacological HO activation attenuates the progression of post-MI LV dysfunction [60] and reduces cardiac inflammation [61]. Most importantly, another orally active EET analog EET-A prevented the development of obesity-induced cardiomyopathy by enhancing HO-1 signaling in mice [58]. Overall, our findings demonstrate that HO-1 is a contributing factor for EET-B mediated reduction of post-MI LV systolic dysfunction and CHF progression in SHR.

In summary, the present study examined the cardio-protective potential of a novel orally active EET analog EET-B in SHR with Mi-induced CHF. EET-B demonstrated cardio-protective actions with lower incidence and severity of ischemic ventricular arrhythmias, reduced CHF-induced lung edema, LV systolic dysfunction, myocardial fibrosis and inflammation in MI-induced CHF model. Our data also suggest that HO-1 signaling can mediates EET-B cardio-protective actions in SHR with Mi-induced CHF.

## Figures and Tables

**Table 1:** Premature ventricle complexes (PVCs) occurring as singles, salvos and ventricular tachycardia (VT), incidence of VT and ventricular fibrillation (VF) and duration of tachyarrhythmias (VT+VF) during 30-min of coronary artery occlusion in vehicle- and EET-B-treated hearts of SHR.

Group			Number of arrhythmias			Incidence (%)		Duration of tachyarrhythmias (s)
	n	Singles	Salvos	VT	PVCs	VT	VF (VFs)	
Vehicle	12	246(250)	167(187)	1750(3538)	2074(3310)	100	61.5 (7.7)	154(304)
EET-B	10	178(338)	93(147)	1240(2585)	1511(2383)	100	77.0 (0)	142(276)

n, number of animals; VFs, sustained VF; values are median (range).

**Table 2:** Echocardiographic parameters of left ventricle in vehicle- and EET-B-treated SHR subjected to myocardial infarction (MI) or sham operation before start of treatment (Baseline), after 10 days of treatment (Before MI) and 7 weeks after MI (End of the study).

	*Baseline*		*Before MI*		*End of the study*			
	Vehicle	EET-B	Vehicle	EET-B	Sham-Vehicle	Sham-EET-B	MI-Vehicle	MI-EET-B
AWTd (mm)	1.78 ± 0.04	1.93 ± 0.06	1.88 ± 0.05	1.98 ± 0.06	1.88 ± 0.09	2.02 ± 0.07	1.85 ± 0.14	1.94 ± 0.18
LVDd (mm)	7.32 ± 0.07	7.11 ± 0.12	7.36 ± 0.07	7.32 ± 0.09	8.16 ± 0.03^‡^	8.16 ± 0.09^‡^	9.60 ± 0.23*	9.49 ± 0.15*
PWTd (mm)	1.83 ± 0.02	1.82 ± 0.05	1.91 ± 0.04	1.91 ± 0.05	1.99 ± 0.07	1.98 ± 0.04	1.94 ± 0.08	2.06 ± 0.10
AWTs (mm)	2.90 ± 0.05	2.94 ± 0.05	3.02 ± 0.06	3.11 ± 0.07	2.96 ± 0.09	3.12 ± 0.06	2.34 ± 0.23*	2.72 ± 0.30
LVDs (mm)	4.67 ± 0.07	4.74 ± 0.12	4.67 ± 0.07	4.67 ± 0.11	5.67 ± 0.10^‡^	5.77 ± 0.12^‡^	7.85 ± 0.26*	7.25 ± 0.15*^†^
PWTs (mm)	2.73 ± 0.04	2.66 ± 0.07	2.86 ± 0.06	2.85 ± 0.06	2.86 ± 0.06	2.87 ± 0.11	2.56 ± 0.07*	2.81 ± 0.14
RWT (%)	49.4 ± 1.0	53.0 ± 1.5	51.6 ± 1.5	53.4 ± 1.6	47.4 ± 1.9	48.9 ± 1.1	40.1 ± 3.1*	42.1 ± 2.2*
HR (bpm)	338 ± 7	308 ± 8	339 ± 9	350 ± 5	355 ± 9.2	337 ± 7	318 ± 7	332 ± 11

AWTd, diastolic anterior wall thickness; LVDd, diastolic left ventricle diameter; PWTd, diastolic posterior wall thickness; AWTs, systolic anterior wall thickness; LVDs, systolic left ventricle diameter; PWTs, systolic posterior wall thickness; RWT, relative wall thickness; HR, heart rate; bpm, beats per minute. Values are means ± SEM from 6-10 rats in each group.

* *P*<0.05 vs. corresponding Sham group;

† *P*<0.05 MI-EET-B vs. MI-Vehicle group.

‡ *P*<0.05 vs. corresponding Before MI group.

**Table 3:** Relative weights of lung and heart 7 wks after MI in vehicle- and EET-B-treated SHR subjected to myocardial infarction (MI) and sham-operated.

	Lungs/BW (mg/g)	Heart/BW (mg/g)
Sham-Vehicle	3.58 ± 0.14	3.57 ± 0.02
Sham-EET-B	3.67 ± 0.09	3.47 ± 0.08
MI-Vehicle	6.28 ± 0.49*	3.94 ± 0.07*
MI-EET-B	4.81 ± 0.49*^†^	3.93 ± 0.12*

Values are means ± SEM from 6-10 rats in each group.

* *P*<0.05 vs. corresponding Sham group;

† *P*<0.05 MI-EET-B group vs. MI-Vehicle group.

## Perspectives

- A novel EET analog EET-B improved cardiac functions independent of blood pressure in SHR with MI induced CHF.
- EET-B beneficial actions to improve LV systolic function in SHR with CHF is associated with increased cardiomyocyte HO-1 levels and reductions in cardiac fibrosis and inflammation.
- EET analog has beneficial cardiac actions and therapeutic potential for CHF.

## ABREVIATIONS

AWTd: diastolic anterior wall thickness
AWTs: systolic anterior wall thickness
ΔAWT: systolic thickening of anterior wall
bpm: beats per minute
BW: body weight
CHF: congestive heart failure
DHETs: dihydroxyeicosatrienoic acids
14,15-DHET: 14,15-dihydroxyeicosatrienoic acid
ECHO: echocardiography
EETs: epoxyeicosatrienoic acids
14,15-EET: 14,15-epoxyeicosatrienoic acid
EF: ejection fraction
FS: fractional shortening
HO-1: heme oxygenase-1
HR: heart rate
LAD: left anterior descending coronary artery
LV: left ventricle
LVDd: diastolic left ventricle diameter
LVDs: systolic left ventricle diameter
MI: myocardial infarction
PVCs: premature ventricle complexes
PWTd: diastolic posterior wall thickness
PWTs: systolic posterior wall thickness
RWT: relative wall thickness
SBP: systolic blood pressure
sEH: soluble epoxide hydrolase
SHR: spontaneously hypertensive rats
SS: salt-sensitive
VF: ventricular fibrillation
VT: ventricular tachycardia.

## References

[1] Roger VL (2013) Epidemiology of heart failure. Circ. Res 113, 646–59 23989710 10.1161/CIRCRESAHA.113.300268 PMC3806290

[2] Imig JD (2012) Epoxides and soluble epoxide hydrolase in cardiovascular physiology. Physiol. Rev 92, 101–30 22298653 10.1152/physrev.00021.2011 PMC3613253

[3] Oni-Orisan A, Alsaleh N, Lee CR and Seubert JM (2014) Epoxyeicosatrienoic acids and cardioprotection: the road to translation. J. Mol. Cell. Cardiol 74, 199–208 24893205 10.1016/j.yjmcc.2014.05.016 PMC4115045

[4] Batchu SN, Lee SB, Samokhvalov V, Chaudhary KR, El-Sikhry H, Weldon SM (2012) Novel soluble epoxide hydrolase inhibitor protects mitochondrial function following stress. Can. J. Physiol. Pharmacol 90, 811–23 22624559 10.1139/y2012-082

[5] Chaudhary KR, Abukhashim M, Hwang SH, Hammock BD and Seubert JM (2010) Inhibition of soluble epoxide hydrolase by trans-4-[4-(3-adamantan-1-yl-ureido)-cyclohexyloxy]-benzoic acid is protective against ischemia-reperfusion injury. J. Cardiovasc. Pharmacol 55, 67–73 19834332 10.1097/FJC.0b013e3181c37d69 PMC2824072

[6] Motoki A, Merkel MJ, Packwood WH, Cao Z, Liu L, Iliff J (2008) Soluble epoxide hydrolase inhibition and gene deletion are protective against myocardial ischemia-reperfusion injury in vivo. Am. J. Physiol. Heart Circ. Physiol 295, H2128–34 18835921 10.1152/ajpheart.00428.2008 PMC2614571

[7] Neckář J, Kopkan L, Husková Z, Kolář F, Papoušek F, Kramer HJ (2012) Inhibition of soluble epoxide hydrolase by cis-4-[4-(3-adamantan-1-ylureido)cyclohexyl-oxy]benzoic acid exhibits antihypertensive and cardioprotective actions in transgenic rats with angiotensin II-dependent hypertension. Clin. Sci. (Lond) 122, 513–25 22324471 10.1042/CS20110622 PMC3528350

[8] Kompa AR, Wang BH, Xu G, Zhang Y, Ho PY, Eisennagel S (2013) Soluble epoxide hydrolase inhibition exerts beneficial anti-remodeling actions post-myocardial infarction. Int. J. Cardiol 167, 210–9 22236509 10.1016/j.ijcard.2011.12.062

[9] Li L, Li N, Pang W, Zhang X, Hammock BD, Ai D (2014) Opposite effects of gene deficiency and pharmacological inhibition of soluble epoxide hydrolase on cardiac fibrosis. PLoS One 9,e94092 24718617 10.1371/journal.pone.0094092 PMC3981766

[10] Merabet N, Bellien J, Glevarec E, Nicol L, Lucas D, Remy-Jouet I (2012) Soluble epoxide hydrolase inhibition improves myocardial perfusion and function in experimental heart failure. J. Mol. Cell. Cardiol 52, 660–6 22155238 10.1016/j.yjmcc.2011.11.015

[11] Sirish P, Li N, Liu JY, Lee KS, Hwang SH, Qiu H (2013) Unique mechanistic insights into the beneficial effects of soluble epoxide hydrolase inhibitors in the prevention of cardiac fibrosis. Proc. Natl. Acad. Sci. U S A 110, 5618–23 23493561 10.1073/pnas.1221972110 PMC3619365

[12] Alánová P, Husková Z, Kopkan L, Sporková A, Jíchová Š, Neckář J (2015) Orally active epoxyeicosatrienoic acid analog does not exhibit antihypertensive and reno- or cardioprotective actions in two-kidney, one-clip Goldblatt hypertensive rats. Vascul. Pharmacol 73, 45–56 26304700 10.1016/j.vph.2015.08.013

[13] Batchu SN, Lee SB, Qadhi RS, Chaudhary KR, El-Sikhry H, Kodela R (2011) Cardioprotective effect of a dual acting epoxyeicosatrienoic acid analogue towards ischaemia reperfusion injury. Br. J. Pharmacol 162, 897–907 21039415 10.1111/j.1476-5381.2010.01093.x PMC3042200

[14] Neckář J, Hsu A, Hye Khan MA, Gross GJ, Nithipatikom K, Cyprová M (2018) Infarct size-limiting effect of epoxyeicosatrienoic acid analog EET-B is mediated by hypoxia inducible factor-1α via down regulation of prolyl hydroxylase 3. Am. J. Physiol. Heart Circ. Physiol 315, H1148–58 30074840 10.1152/ajpheart.00726.2017 PMC6734065

[15] Hye Khan MA, Neckář J, Manthati V, Errabelli R, Pavlov TS, Staruschenko A (2013) Orally active epoxyeicosatrienoic acid analog attenuates kidney injury in hypertensive Dahl salt-sensitive rat. Hypertension 62, 905–13 23980070 10.1161/HYPERTENSIONAHA.113.01949 PMC3872985

[16] Hye Khan MA, Pavlov TS, Christain SV, Neckář J, Staruschenko A, Gauthier KM (2014) Epoxyeicosatrienoic acid analogue lowers blood pressure through vasodilation and sodium channel inhibition. Clin. Sci. (Lond) 127: 463–74 24707975 10.1042/CS20130479 PMC4167712

[17] Yeboah MM, Hye Khan MA, Chesnik MA, Sharma A, Paudyal MP, Falck JR (2016) The epoxyeicosatrienoic acid analog PVPA ameliorates cyclosporine-induced hypertension and renal injury in rats. Am. J. Physiol. Renal Physiol 311, F576–85 27358055 10.1152/ajprenal.00288.2016 PMC5504406

[18] Cao J, Tsenovoy PL, Thompson EA, Falck JR, Touchon R, Sodhi K (2015) Agonists of epoxyeicosatrienoic acids reduce infarct size and ameliorate cardiac dysfunction via activation of HO-1 and Wnt1 canonical pathway. Prostaglandins Other Lipid Mediat 116-117, 76–86 25677507 10.1016/j.prostaglandins.2015.01.002 PMC5553685

[19] Červenka L, Husková Z, Kopkan L, Kikerlová S, Sedláková L, Vaňourková Z (2018) Two pharmacological epoxyeicosatrienoic acid-enhancing therapies are effectively antihypertensive and reduce the severity of ischemic arrhythmias in rats with angiotensin II-dependent hypertension. J. Hypertens 36, 1326–41 29570510 10.1097/HJH.0000000000001708 PMC7375140

[20] Singh SP, Bellner L, Vanella L, Cao J, Falck JR, Kappas A (2016) Downregulation of PGC-1α prevents the beneficial effect of EET-heme oxygenase-1 on mitochondrial integrity and associated metabolic function in obese mice. J. Nutr. Metab 2016, 9039754 28097021 10.1155/2016/9039754 PMC5206458

[21] Chandler MP and DiCarlo SE (1998) Arterial baroreflex resetting mediates postexercise reductions in arterial pressure and heart rate. Am. J. Physiol 275, H1627–34 9815070 10.1152/ajpheart.1998.275.5.H1627

[22] Romashko M, Schragenheim J, Abraham NG and McClung JA (2016) Epoxyeicosatrienoic acid as therapy for diabetic and ischemic cardiomyopathy. Trends Pharmacol. Sci 37, 945–962 27633970 10.1016/j.tips.2016.08.001

[23] Imig JD, Falck JR, Campbell WB (2015) Epoxyeicosatrienoic acid analogs and methods of making and using the same. U.S. Patent 9, 127, 027 B2

[24] Imig JD, Falck JR, Campbell WB (2016) Epoxyeicosatrienoic acid analogs and methods of making and using the same. U.S. Patent 9, 422, 318

[25] Falck JR, Koduru SR, Mohapatra S, Manne R, Atcha KR, Manthati VL, (2014) Robust surrogates of 14,15-epoxyeicosa-5,8,11-trienoic acid (14,15-EET): carboxylate modifications. Med. Chem 57, 6965–72 10.1021/jm500262m PMC4148164 25119815

[26] Neckář J, Papoušek F, Nováková O, Oštádal B and Kolář F (2002) Cardioprotective effects of chronic hypoxia and ischaemic preconditioning are not additive. Basic Res. Cardiol 97, 161–7 12002264 10.1007/s003950200007

[27] Asemu G, Neckář J, Szárszoi O, Papousek F, Ostádal B, Kolář F (2000) Effects of adaptation to intermittent high altitude hypoxia on ischemic ventricular arrhythmias in rats. Physiol. Res 49, 597–606. 11191364

[28] Imig JD and Hammock BD (2009) Soluble epoxide hydrolase as a therapeutic target for cardiovascular diseases. Nat. Rev. Drug Discov 8, 794–805 19794443 10.1038/nrd2875 PMC3021468

[29] Luo P, Chang HH, Zhou Y, Zhang S, Hwang SH, Morisseau C (2010) Inhibition or deletion of soluble epoxide hydrolase prevents hyperglycemia, promotes insulin secretion, and reduces islet apoptosis. J. Pharmacol. Exp. Ther 334, 430–8 20439437 10.1124/jpet.110.167544 PMC2913776

[30] Roche C, Besnier M, Cassel R, Harouki N, Coquerel D, Guerrot D (2015) Soluble epoxide hydrolase inhibition improves coronary endothelial function and prevents the development of cardiac alterations in obese insulin-resistant mice. Am. J. Physiol. Heart Circ. Physiol 308, H1020–9 25724490 10.1152/ajpheart.00465.2014 PMC4551118

[31] Pozzi A, Macias-Perez I, Abair T, Wei S, Su Y, Zent R (2005) Characterization of 5,6- and 8,9-epoxyeicosatrienoic acids (5,6- and 8,9-EET) as potent in vivo angiogenic lipids. J. Biol.Chem 280, 27138–46 15917237 10.1074/jbc.M501730200

[32] Xu DY, Davis BB, Wang ZH, Zhao SP, Wasti B, Liu ZL (2013) A potent soluble epoxide hydrolase inhibitor, t-AUCB, acts through PPARγ to modulate the function of endothelial progenitor cells from patients with acute myocardial infarction. Int. J. Cardiol 167, 1298–304 22525341 10.1016/j.ijcard.2012.03.167 PMC3821736

[33] Gross GJ, Baker JE, Hsu A, Wu HE, Falck JR, Nithipatikom K (2008) Effects of the selective EET antagonist, 14,15-EEZE, on cardioprotection produced by exogenous or endogenous EETs in the canine heart. Am. J. Physiol. Heart Circ. Physiol 294, H2838–44 18441205 10.1152/ajpheart.00186.2008 PMC2863006

[34] Gross GJ, Hsu A, Pfeiffer AW and Nithipatikom K (2013) Roles of endothelial nitric oxide synthase (eNOS) and mitochondrial permeability transition pore (MPTP) in epoxyeicosatrienoic acid (EET)-induced cardioprotection against infarction in intact rat hearts. J. Mol. Cell. Cardiol 59, 20–9 23419451 10.1016/j.yjmcc.2013.02.003 PMC3647061

[35] Batchu SN, Lee SB, Qadhi RS, Chaudhary KR, El-Sikhry H, Kodela R (2011) Cardioprotective effect of a dual acting epoxyeicosatrienoic acid analogue towards ischaemia reperfusion injury. Br. J. Pharmacol 162, 897–907 21039415 10.1111/j.1476-5381.2010.01093.x PMC3042200

[36] Khan MA, Liu J, Kumar G, Skapek SX, Falck JR, Imig JD (2013) Novel orally active epoxyeicosatrienoic acid (EET) analogs attenuate cisplatin nephrotoxicity. FASEB J 27, 2946–56 23603837 10.1096/fj.12-218040 PMC3714587

[37] Ai D, Pang W, Li N, Xu M, Jones PD, Yang J (2009) Soluble epoxide hydrolase plays an essential role in angiotensin II-induced cardiac hypertrophy. Proc. Natl. Acad. Sci. U S A 106, 564–9 19126686 10.1073/pnas.0811022106 PMC2626743

[38] Jíchová Š, Kopkan L, Husková Z, Doleželová Š, Neckář J, Kujal P (2016) Epoxyeicosatrienoic acid analog attenuates the development of malignant hypertension, but does not reverse it once established: a study in Cyp1a1-Ren-2 transgenic rats. J. Hypertens 34, 2008–25 27428043 10.1097/HJH.0000000000001029 PMC5510029

[39] Kujal P, Čertíková Chábová V, Śkaroupková P, Husková Z, Vernerová Z, Kramer HJ (2014) Inhibition of soluble epoxide hydrolase is renoprotective in 5/6 nephrectomized Ren-2 transgenic hypertensive rats. Clin. Exp. Pharmacol. Physiol 41, 227–37 24471737 10.1111/1440-1681.12204 PMC4038339

[40] Monti J, Fischer J, Paskas S, Heinig M, Schulz H, Gösele C (2008) Soluble epoxide hydrolase is a susceptibility factor for heart failure in a rat model of human disease. Nat. Genet 40, 529–37 18443590 10.1038/ng.129 PMC7370537

[41] Wang X, Ni L, Yang L, Duan Q, Chen C, Edin ML, Zeldin DC (2014) CYP2J2-derived epoxyeicosatrienoic acids suppress endoplasmic reticulum stress in heart failure. Mol. Pharmacol 85, 105–15 24145329 10.1124/mol.113.087122 PMC3868901

[42] Xu D, Li N, He Y, Timofeyev V, Lu L, Tsai HJ (2006) Prevention and reversal of cardiac hypertrophy by soluble epoxide hydrolase inhibitors. Proc. Natl. Acad. Sci. U S A 103, 18733–8 17130447 10.1073/pnas.0609158103 PMC1693731

[43] Shen HC, Ding FX, Deng Q, Xu S, Chen HS, Tong X (2009) Discovery of 3,3-disubstituted piperidine-derived trisubstituted ureas as highly potent soluble epoxide hydrolase inhibitors. Bioorg. Med. Chem. Lett 19, 5314–20 19682899 10.1016/j.bmcl.2009.07.138

[44] Khan AH, Falck JR, Manthati VL, Campbell WB and Imig JD (2014) Epoxyeicosatrienoic acid analog attenuates angiotensin II hypertension and kidney injury. Front. Pharmacol 5, 216 25295006 10.3389/fphar.2014.00216 PMC4172029

[45] Braunwald E (2015) The war against heart failure: the Lancet lecture. Lancet 385, 812–24 25467564 10.1016/S0140-6736(14)61889-4

[46] Zhang K, Liu Y, Liu X, Chen J, Cai Q, Wang J (2015) Apocynin improving cardiac remodeling in chronic renal failure disease is associated with up-regulation of epoxyeicosatrienoic acids. Oncotarget 6, 24699–708 26322503 10.18632/oncotarget.5084 PMC4694789

[47] Schuck RN, Theken KN, Edin ML, Caughey M, Bass A, Ellis K (2013) Cytochrome P450-derived eicosanoids and vascular dysfunction in coronary artery disease patients. Atherosclerosis 227, 442–8 23466098 10.1016/j.atherosclerosis.2013.01.034 PMC3638946

[48] Akhnokh MK, Yang FH, Samokhvalov V, Jamieson KL, Cho WJ, Wagg C (2016) Inhibition of soluble epoxide hydrolase limits mitochondrial damage and preserves function following ischemic injury. Front. Pharmacol 7, 133 27375480 10.3389/fphar.2016.00133 PMC4896112

[49] Hrdlička J, Neckář J, Papoušek F, Husková Z, Kikerlová S, Vańourková Z (2019) Epoxyeicosatrienoic acid-based therapy attenuates the progression of postischemic heart failure in normotensive Sprague-Dawley but not in hypertensive *Ren-2* transgenic rats. Front. Pharmacol 10, 159 30881303 10.3389/fphar.2019.00159 PMC6406051

[50] Morgan LA, Olzinski AR, Upson JJ, Zhao S, Wang T, Eisennagel SH (2013). Soluble epoxide hydrolase inhibition does not prevent cardiac remodeling and dysfunction after aortic constriction in rats and mice. J. Cardiovasc. Pharmacol 61, 291–301 23232840 10.1097/FJC.0b013e31827fe59c

[51] Červenka L, Melenovský V, Husková Z, Škaroupková P, Nishiyama A, and Sadowski J (2015). Inhibition of soluble epoxide hydrolase counteracts the development of renal dysfunction and progression of congestive heart failure in Ren-2 transgenic hypertensive rats with aorto-caval fistula. Clin. Exp. Pharmacol. Physiol 42, 795–807 25969338 10.1111/1440-1681.12419

[52] Červenka L, Melenovský V, Husková Z, Sporková A, Bürgelová M, Škaroupková P (2015). Inhibition of soluble epoxide hydrolase does not improve the course of congestive heart failure and the development of renal dysfunction in rats with volume overload induced by aorto-caval fistula. Physiol. Res 64, 857–73 26047375 10.33549/physiolres.932977 PMC4984848

[53] Vacková Š, Kopkan L, Kikerlová S, Husková Z, Sadowski J, Kompanowska-Jezierska E (2019). Pharmacological blockade of soluble epoxide hydrolase attenuates the progression of congestive heart failure combined with chronic kidney disease: insights from studies with Fawn-hooded hypertensive rats. Front. Pharmacol 10, 18 30728778 10.3389/fphar.2019.00018 PMC6351500

[54] He Z, Zhang X, Chen C, Wen Z, Hoopes SL, Zeldin DC (2015) Cardiomyocyte-specific expression of CYP2J2 prevents development of cardiac remodelling induced by angiotensin II. Cardiovasc. Res 105, 304–17 25618409 10.1093/cvr/cvv018 PMC4351370

[55] Xiao B, Li X, Yan J, Yu X, Yang G, Xiao X (2010) Overexpression of cytochrome P450 epoxygenases prevents development of hypertension in spontaneously hypertensive rats by enhancing atrial natriuretic peptide. J. Pharmacol. Exp. Ther 334, 784–94 20501636 10.1124/jpet.110.167510 PMC2939659

[56] Hutchens MP, Nakano T, Dunlap J, Traystman RJ, Hurn PD, Alkayed NJ (2008) Soluble epoxide hydrolase gene deletion reduces survival after cardiac arrest and cardiopulmonary resuscitation. Resuscitation 76, 89–94 17728042 10.1016/j.resuscitation.2007.06.031 PMC2585367

[57] Sacerdoti D, Pesce P, Di Pascoli M and Bolognesi M (2016) EETs and HO-1 cross-talk. Prostaglandins Other Lipid Mediat 125, 65–79 27354356 10.1016/j.prostaglandins.2016.06.002

[58] Cao J, Singh SP, McClung JA, Joseph G, Vanella L, Barbagallo I (2017) EET intervention on Wnt1, NOV, and HO-1 signaling prevents obesity-induced cardiomyopathy in obese mice. Am. J. Physiol. Heart Circ. Physiol 313, H368–H380 28576832 10.1152/ajpheart.00093.2017 PMC5582926

[59] Otterbein LE, Foresti R and Motterlini R (2016) Heme oxygenase-1 and carbon monoxide in the heart: the balancing act between danger signaling and pro-survival. Circ. Res 118, 1940–1959 27283533 10.1161/CIRCRESAHA.116.306588 PMC4905590

[60] Chen T, Li J, Liu L, Fan L, Li XY, Wang YT (2013) Effects of heme oxygenase-1 upregulation on blood pressure and cardiac function in an animal model of hypertensive myocardial infarction. Int. J. Mol. Sci 14, 2684–706 23358254 10.3390/ijms14022684 PMC3588009

[61] Elmarakby AA, Faulkner J, Posey SP and Sullivan JC (2010) Induction of heme oxygenase-1 attenuates the hypertension and renal inflammation in spontaneously hypertensive rats. Pharmacol. Res 62, 400–407 20667508 10.1016/j.phrs.2010.07.005

